# A Critical Review: Gel-Based Edible Inks for 3D Food Printing: Materials, Rheology–Geometry Mapping, and Control

**DOI:** 10.3390/gels11100780

**Published:** 2025-09-29

**Authors:** Zhou Qin, Yang Yang, Zhaomin Zhang, Fanfan Li, Ziqing Hou, Zhihua Li, Jiyong Shi, Tingting Shen

**Affiliations:** 1 Agricultural Product Processing and Storage Lab, School of Food and Biological Engineering, Jiangsu University, Zhenjiang 212013, Chinalff1067524252@gmail.com (F.L.);; 2 International Joint Research Laboratory of Intelligent Agriculture and Agro-Products Processing, Jiangsu Education Department, Zhenjiang 212013, China

**Keywords:** gels, 3D food printing, edible hydrogels, extrusion, rheology, printability, emulsion

## Abstract

Edible hydrogels are the central material class in 3D food printing because they reconcile two competing needs: (i) low resistance to flow under nozzle shear and (ii) fast recovery of elastic structure after deposition to preserve geometry. This review consolidates the recent years of progress on hydrogel formulations—gelatin, alginate, pectin, carrageenan, agar, starch-based gels, gellan, and cellulose derivatives, xanthan/konjac blends, protein–polysaccharide composites, and emulsion gels alongside a critical analysis of printing technologies relevant to food: extrusion, inkjet, binder jetting, and laser-based approaches. For each material, this review connects gelation triggers and compositional variables to rheology signatures that govern printability and then maps these to process windows and post-processing routes. This review consolidates a decision-oriented workflow for edible-hydrogel printability that links formulation variables, process parameters, and geometric fidelity through standardized test constructs (single line, bridge, thin wall) and rheology-anchored gates (e.g., yield stress and recovery). Building on these elements, a “printability map/window” is formalized to position inks within actionable operating regions, enabling recipe screening and process transfer. Compared with prior reviews, the emphasis is on decisions: what to measure, how to interpret it, and how to adjust inks and post-set enablers to meet target fidelity and texture. Reporting minima and a stability checklist are identified to close the loop from design to shelf.

## 1. Introduction

The spatial architecture of a meal, its texture, porosity, and kinetics of nutrient release can now be specified with the precision of a digital design [1,2]. Gel-based three-dimensional food printing enables this capability by converting food-grade hydrocolloids into engineered textures. These include textures that support safe swallowing in clinical nutrition, plant-based constructs that emulate animal tissue, and visually intricate constructions with controlled sugar migration [3,4,5]. And visually intricate confections with controlled sugar migration. Because gels are water-rich, biocompatible, and thermodynamically versatile [6], they intersect rheology, soft-matter physics, human nutrition, and manufacturing science, positioning edible hydrogels as the functional core of three-dimensional food printing rather than a passive carrier [7].

Across three-dimensional food printing, extrusion remains the predominant method for depositing viscoelastic printing inks with controlled strand width and shape fidelity, whereas jetting, binder-based, and laser processes are used primarily for powders and sugars [8,9,10]. Within this ecosystem, edible hydrogels—including gelatin, alginate, pectin (low-methoxyl and high-methoxyl), carrageenan, agar, starches, gellan, methylcellulose, xanthan, konjac glucomannan, and protein–polysaccharide co-gelled networks—are widely used. These materials provide orthogonal gelation triggers (thermal, ionic, and dehydration routes) and tunable interpenetrating networks that decouple printability from final textural targets [11,12,13]. Prior studies show that a compact set of rheological descriptors near the build temperature—yield stress, storage modulus, and shear-thinning index—predicts strand stand-up, wall-angle retention, and filament continuity across multiple ink families. Recent studies emphasize pragmatic “printability windows,” in which combinations of storage modulus, loss tangent, and apparent viscosity enable high line fidelity without nozzle clogging or die swell artifacts [14,15]. At the product level, applications concentrate on four domains: dysphagia-oriented soft textures for safe swallowing, low-sugar fruit gels with controllable syneresis, plant-based structured foods with graded anisotropy, and emulsion gels as carriers for flavors, probiotics, and micronutrients. In parallel, in situ imaging, compact print-quality benchmarks, and early data-driven or inverse-design tools are beginning to link formulation, rheology, tool path, and post-setting conditioning [16].

**Figure 1:**
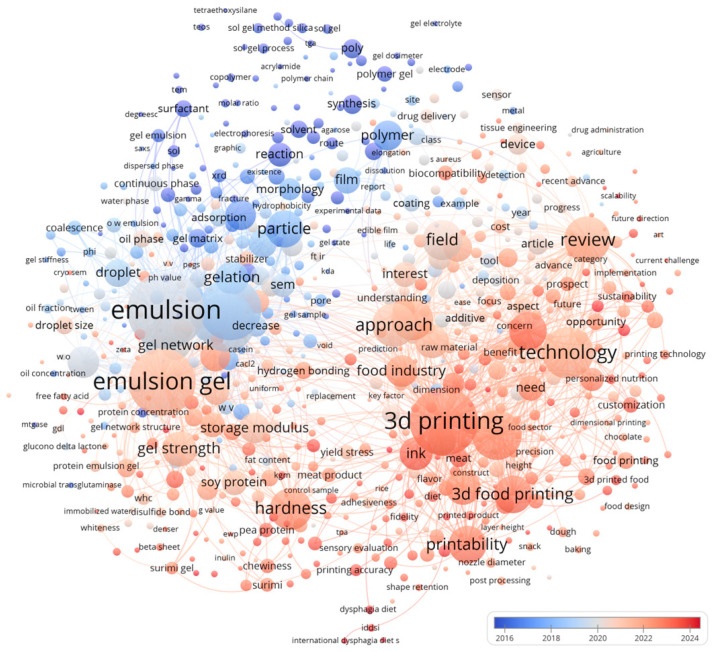
Research status of edible gels and emulsions in 3D food printing.

Edible-hydrogel printing matters across three fronts. For the industry, it enables mass personalization, rapid iteration of textures and shapes, and integration with existing forming lines. For nutrition and public health, gel-forward formulations support texture-modified diets, portion control, and controlled delivery of bioactives. For consumers, printed foods can improve acceptability, enable soft-solid formats that are attractive and safe, and create on-demand experiences without compromising ingredient lists [17,18]. Figure 1 reveals two primary clusters. The Materials/Colloids cluster is organized around “emulsion” and “emulsion gel” and is closely associated with gelation, droplet size, gel network, storage modulus, gel strength, and polymer chemistry terms. The Engineering/Applications cluster centers on “three-dimensional printing,” “technology,” “printability,” and “ink,” radiating to customization, the food industry, plant-based proteins (soy, pea, and surimi), dysphagia, hardness, and sensory evaluation [19,20,21,22]. Cross-cluster bridging through rheological descriptors—yield stress, storage modulus, and indentation hardness—indicates that mechanical metrics mediate the translation from formulation to tool path and product properties. Interest in printing-centric topics is increasing. By contrast, relatively sparse connections to post-processing, shelf life, and packaging suggest limited integration of later-stage considerations. Overall, the network depicts edible hydrogels and emulsion gels as the material pillars, with “printability” serving as the operational hub for applications.

Despite this progress, three barriers limit translation and comparability. First, methods remain heterogeneous: many studies emphasize steady-shear data while undersampling time–temperature–salt histories and yield-dominated regimes that govern strand stability, leading to ad hoc “printable/non-printable” labels with poor external validity. Second, cross-material generalization is weak; frameworks developed for a single gel family rarely transfer to blends or co-networks in which protein–polysaccharide interactions introduce hidden variables (for example, counter-ion identity and charge density) that shift the printability window and the sensory profile. Third, post-setting enablers—ionic curing, thermal annealing, dehydration, and packaging—are often treated as secondary, even though shelf life, water activity, and mechanical stability ultimately determine product viability and safety. Consequently, the field lacks (i) a decision-oriented synthesis that maps materials to processes and post-setting routes, (ii) standardized, small-footprint print-quality metrics that correlate with rheology, and (iii) a best-practice workflow that integrates formulation screening, tool-path selection, and storage design. Although individual reports address specific elements, a consolidated, cross-family framework that is actionable for both researchers and practitioners is still missing.

This review addresses these gaps through a decision-centric synthesis organized around materials-to-process matching and validated by quantitative print-quality measures. Specifically, this review synthesizes rheological predictors of printability across major edible gel families and formalizes pragmatic printability windows measurable at the build temperature. Benchmarks compact, image-based print-quality metrics against those rheological descriptors to establish transferable pass–fail criteria. Maps gelation pathways (thermal, ionic, and dehydration) to post-setting enablers for shelf-life control, highlighting trade-offs among syneresis, mechanical integrity, and sensory targets. Consolidates these insights into a field-usable printability map and an eight-step best-practice workflow that moves beyond trial-and-error tuning. Identifies forward paths in which in situ sensing and data-driven inverse design reduce formulation search space and improve reproducibility. Our guiding hypothesis is that a minimal set of rheological and thermal-kinetic descriptors, paired with standardized print-quality scores, generalizes across gel families and printing modalities sufficiently to support reliable material-process decisions. By aligning soft-matter principles with constraints from clinical nutrition, plant-based foods, and confectionery manufacturing, this framework aims to accelerate safe, scalable, and nutritionally meaningful gel-forward products.

## 2. Printability Fundamentals and Characterization

### 2.1. Rheological Predictors of Printability

For edible hydrogel inks, the descriptors that best predict printability are those directly tied to in-nozzle flow, post-deposition structural recovery, and geometric fidelity. In practice, representing the flow curve at the build temperature with the Herschel–Bulkley model—quantified by the apparent yield stress, the consistency index, and a flow index less than one—captures whether a given ink will initiate and sustain filament formation under device-limited pressure. Mapping these parameters onto the nozzle-relevant shear-rate band (on the order of 10^2^ to 10^4^ per second, estimated from the inner diameter and volumetric flow) transforms rheometer measurements into pressure–speed set points rather than generic viscosity values. Outside the nozzle, a three-interval thixotropy test or an equivalent step-shear protocol yields a characteristic recovery time (for example, the time to recover eighty percent of the pre-shear modulus) that correlates with corner sharpness, stringing, and sag. A small-strain linear viscoelastic frequency sweep at the build temperature, verifying a storage modulus greater than the loss modulus in the one to ten hertz range, indicates a solid-dominated network capable of supporting strands and overhangs. Elasticity at the nozzle exit manifests as die swell; reporting the swell ratio, defined as the strand diameter divided by the nozzle diameter, provides a geometric proxy for elastic recoil at the nozzle tip [23,24].

Rheometry and texture analysis probe different but overlapping aspects of printability, and both are susceptible to protocol-driven variance that limits inter-lab transferability. In rheometry, the combined effects of geometry choice and surface roughness (smooth versus serrated plates), wall slip, gap setting, edge truncation, temperature drift and equilibration, pre-shear/rest history, and the selection of strain amplitude and frequency can materially alter estimated yield stress (τ_y_), storage modulus (G′), and recovery behavior. Small deviations in these anchors often exceed the differences between formulations, which explains contradictory trends reported across laboratories. Texture methods (compression, TPA-like protocols, back-extrusion) likewise depend strongly on probe geometry and speed, sample height/diameter, surface lubrication, and the rest time allowed for structural relaxation; as a result, “hardness,” “cohesiveness,” and “extrusion force” are not absolute material constants but conditional measures tied to fixture and boundary conditions.

To enable reproducible comparison, minimum reporting should, at a minimum, specify for rheometry: geometry and surface roughness, gap and truncation method, temperature setpoint and equilibration time, pre-shear magnitude/duration and rest time, and the chosen strain/frequency relative to the linear viscoelastic range; and for texture tests: probe type/diameter and speed, sample dimensions and fill height, surface condition, and rest time before testing. Replicates and a repeatability metric are recommended. Crucially, both rheological and texture readouts should be mapped to printing functions, strand stability, bridge retention, strand-to-strand fusion, and shape fidelity, so that decisions emphasize functional thresholds rather than nominal absolute values.

### 2.2. Printability Windows: Parameter Ranges and Decision Criteria

Recent studies converge on pragmatic operating windows that balance pressure limits, ooze control, and geometric fidelity for soft, edible structures. Yield levels in the tens to a few hundreds of pascals at the build temperature suppress dripping while keeping syringe pressures feasible; taller, self-supporting structures typically require yields in the low-kilopascal range. Pronounced shear-thinning behavior (flow index less than one) is necessary to increase printing speed without incurring runaway pressure. After deposition, a storage modulus greater than the loss modulus in the low-hertz band and a structural-recovery time shorter than the inter-pass dwell time improve bridges and overhangs; when the measured swell ratio differs substantially from unity, early locking strategies—such as active cooling or coaxial ionic gelation—are warranted. Studies that report these operating windows together with pressure–flow calibration for the specific device achieve substantially tighter predictions of bead width and dimensional error than approaches that rely on viscosity measurements alone [25,26]. The printability framework translates standardized rheology and texture inputs into functional ranges for strand stability, bridge retention, strand-to-strand fusion, and shape fidelity, which supports cross-laboratory and industrial use under varied equipment and budget constraints.

### 2.3. Quantitative Evaluation of Print Quality

Comparability improves when rheological measurements are coupled with a compact, reproducible set of calibration artifacts and automated computer-vision analysis. Single-wall lines, right-angle turns, fixed-span bridges, thin-wall cylinders, and open lattices reveal control of strand width, corner rounding, sag, interlayer welding, and void formation; recent studies demonstrate that layer-by-layer computer-vision workflows quantify over- and under-extrusion, corner radii, and bridge deflection in ways that correlate with human assessment. Feature-based print-quality scoring frameworks from the three-dimensional food printing literature complement these metrics and enable systematic iteration across formulations and tool paths. Critically, every print-quality metric should be logged with the nozzle identifier, layer height, printing speeds, and the thermal or ionic gelation protocol used, so that couplings among rheology, process conditions, and geometry are learnable and transferable across gel families [27,28]. Some typical cases are shown in Table 1:

Collectively, the surveyed studies outline a convergent framework for quantitatively assessing print quality in gel-based extrusion printing. A compact suite of geometric metrics—line-width error, the area fraction of over- and under-extrusion, bridge deflection, parallel-strand fusion width, corner radius, and layer thickness—measured on standardized calibration artifacts (single line, ninety-degree corner, fixed-span bridge, fusion coupon, thin-wall cylinder, and open-cell lattice) yields reproducible cross-material comparisons and aligns with perceptual ratings, thereby enabling operational pass–fail thresholds.

When linked to rheological measurements at the build temperature, these geometric metrics become predictive. The apparent yield stress, storage modulus, loss tangent, shear-thinning index, and the three-interval thixotropy test correlate with strand stand-up, wall fidelity, and resistance to collapse, permitting the definition of practical “printability windows.” Process metrology further reduces variability: calibration of printing speed, applied pressure, and volumetric flow decreases line-width scatter, and the die swell ratio (that is, the swell ratio) serves as a proxy for elastic recoil at the nozzle exit and an early indicator of dimensional drift. Where rheometry is unavailable, texture analysis provides a useful surrogate for rapid formulation screening.

In situ sensing and data-driven methods extend this framework toward control. Computer vision, optical coherence tomography, and ultrasound enable online estimation of filament width, layer thickness, and inter-layer bonding, whereas design-of-experiments and machine-learning workflows support parameter optimization and cross-formulation generalization. On this basis, a minimal reporting set is recommended: (i) standardized geometric metrics on common artifacts, (ii) rheological parameters at the build temperature, (iii) process proxies (pressure–flow calibration and die swell ratio), and (iv) in situ sensing readouts—implemented within a workflow that proceeds from geometric quantification to rheology-informed interpretation, followed by calibrated deposition and real-time feedback. This structure enhances comparability and transferability across formulations, tool paths, and platforms.

### 2.4. Methodological Limitations and Mitigation Strategies

Current studies still overemphasize steady-shear flow curves while underreporting recovery behavior and elasticity at the nozzle exit, even though the latter dominate corner fidelity, sag, and line thickening; thermal and ionic fields are often described qualitatively rather than logged quantitatively, which impedes cross-laboratory reconstruction of gelation kinetics [38]. Printability claims also remain agnostic to geometry when they rely on “it printed” without standardized calibration artifacts or numeric scores, and device coupling—pressure ceilings, line compliance, and nozzle tolerances—often goes unreported despite its strong influence on extrusion dynamics. Recent studies describe low-cost, immediately adoptable mitigations: include a three-interval thixotropy recovery measurement and a die swell measurement in the core characterization panel; instrument and report syringe-barrel, build-plate, and ambient temperatures, as well as the calcium-ion (Ca^2+^) delivery mode and concentration; and publish images of the calibration artifacts with machine-readable metrics together with a brief pressure–flow calibration for the specific printing system. When swell or slow structural recovery persists, early locking through coaxial ionic gelation and localized cooling reliably stabilizes line width and overhangs without major reformulation, and these strategies are now documented in food-grade formulations [25,35].

### 2.5. Data Infrastructure, In-Situ Sensing, and Inverse Design

Progress in the next phase will depend on curated, shareable data sets that couple rheology, setting kinetics, and geometry-resolved printing outcomes under well-specified thermal or ionic fields. Recent studies have begun to release structured hydrogel printability databases and to learn quantitative relationships among Herschel–Bulkley model fits, structural recovery, and strand-scale fidelity using machine learning, demonstrating that multi-material hydrogel data can support predictive maps rather than heuristic rules [36,39]. These efforts point to a pathway in which inverse design—mapping target bead width and angle retention to feasible composition–process formulations—becomes routine, provided that the community logs comparable features and device states. To anchor such efforts, perspectives and reviews in bioprinting emphasize that “printability windows” must be made operational through transparent reporting and realistic targets; otherwise, the field risks non-transferable claims and overstatement [40].

A second pillar is measurement: image-based accuracy tools and design-feature scoring now quantify layer accuracy, over- and under-extrusion, corner radii, and bridge deflection in ways that correlate with expert assessment and can be automated for throughput; computer vision–based feedback can also adjust tool paths in real time to reduce filament–path mismatch. These approaches supply the standardized calibration artifacts and metrics needed for data-set growth across food and biological hydrogel systems [28,30].

Finally, in situ sensing will close the loop from characterization to control. Optical coherence tomography platforms already enable volumetric, layer-by-layer inspection and defect-aware feedback control during extrusion or Freeform Reversible Embedding of Suspended Hydrogels (FRESH) printing, whereas recent ultrasound implementations provide sub-wavelength monitoring of hydrogel deposition, inter-layer bonding, transient elasticity, and even post-crosslinking in calcium chloride (CaCl_2_) baths, directly informing the timing of coaxial or bath gelation. As these sensing streams are fused with rheology and process logs, lightweight surrogate models can steer early-locking strategies—such as coaxial ion delivery and active cooling—to suppress die swell and stabilize thin features, thereby moving beyond ad hoc tuning [33,34].

## 3. Edible Hydrogel Materials

### 3.1. Gelatin

In gelatin-based inks, small additions of kappa-carrageenan (κ-carrageenan) or gellan gum generate protein–polysaccharide co-networks that increase the early yield stress and the storage modulus at the build temperature [41]. These formulations extrude at practical pressures and produce cleaner filaments with improved strand stand-up and wall-angle retention [42]. In systems based on fish gelatin and high-acyl gellan gum, comparable performance is achieved at modest total solids, which aligns with clean-label objectives in three-dimensional food printing [43]. The principal trade-offs are brittleness and syneresis when networks are over-stiffened or when kappa-carrageenan fractions are high, motivating co-network strategies that decouple in-nozzle flow from post-setting firmness [44]. Low-temperature deposition further “locks” the filament diameter early and suppresses die swell. Looking forward, protein–polysaccharide co-gels tuned by cation identity and controlled thermal fields (for example, cooled nozzles and heated build plates or staged post-heating) should preserve extrudability while delivering the desired post-setting firmness. Coupling these strategies to standardized geometric metrics and storage assays will convert rheological gains into reproducible, shelf-life-aware gelatin inks [45]. Table 2 presents representative applications of gelatin inks in extrusion-based 3D printing.

Small additions of gellan gum or kappa-carrageenan (κ-carrageenan) to gelatin generate protein–polysaccharide co-networks that increase the storage modulus and the yield stress at the build temperature [49], enabling practical extrusion pressures while producing cleaner filaments, superior strand stand-up, and improved wall-angle retention. Systems based on fish gelatin and high-acyl gellan gum achieve these effects at modest total solids, and low-temperature deposition further locks the filament diameter early and suppresses die swell. The benefits, however, depend on the operating window: high kappa-carrageenan fractions or over-stiffened networks increase brittleness and syneresis and undermine shelf life and clean-label objectives, and most reports prioritize immediate geometric fidelity over long-term dimensional stability. Going forward, decoupled co-networks—gelatin combined with low-dose gellan gum or kappa-carrageenan tuned by cation identity—and controlled thermal fields (cooled nozzles, heated build plates, or staged post-heating) should preserve extrudability while delivering the desired post-setting firmness. Coupling these strategies to standardized geometric metrics and storage assays will translate rheological gains into reproducible, shelf-life-aware gelatin inks.

### 3.2. Alginate

Coaxial calcium-ion (Ca^2+^) delivery has become a practical route to immediate strand stand-up and tall builds at relatively low solids [50,51,52], because a thin, rapidly gelled alginate sheath stabilizes the freshly extruded core before substantial die swell or gravity-induced creep occurs [25,53]. Compared with bath curing, coaxial exposure localizes crosslinking at the perimeter, preserving in-nozzle flow while increasing the early yield stress around the strand. Studies report clean filaments, reduced lattice sag, and improved wall-angle retention when the sheath concentration and the sheath-to-core flow ratio are tuned to the deposition speed and nozzle geometry. Blends with low-methoxyl pectin extend this concept: alginate provides rapid ionic “lock-in,” whereas low-methoxyl pectin increases ductility and structural recovery, reducing crack propensity in bending and multilayer builds [38]. Moreover, alginate–pectin shells enable acidity-responsive (pH-responsive) core–shell constructs in which acidic triggers or gastrointestinal cues modulate release, aligning edible three-dimensional printed structures with functional-food objectives beyond shape fidelity alone [54].

Key limitations persist. High calcium-ion activity or prolonged exposure can over-stiffen the shell, generate steep core–shell gradients, and produce brittle, crack-prone filaments with reduced chewability. Diluted sheaths, shorter residence zones, and spatially confined delivery mitigate these effects but narrow the processing window. Internal gelation strategies trade spatial control for slower kinetics, whereas multivalent alternatives (for example, barium ion, Ba^2+^) raise regulatory and sensory concerns. Storage stability remains sensitive to ion exchange and moisture migration, with dimensional drift under chilled conditions when the shell is overly rigid. Future work should formalize dimensionless design rules to generalize across nozzles and climates. Hybrid shells can be engineered for graded crosslinking and programmable ductility, aided by buffered calcium-ion activity, chelators, or staged post-exposure. Finally, in situ sensing paired with standardized geometric metrics should close the loop between ionic flux, rheology at the build temperature, and multilayer fidelity, enabling shelf-life-aware, function-forward alginate systems [55,56].

### 3.3. Pectin

Low-methoxyl pectin addresses low-sugar constraints through calcium-ion-mediated “egg-box” gelation that provides usable yield stress and rapid structural recovery at moderate solids, conditions under which high-methoxyl pectin typically relies on acid and sucrose loading [57,58]. Mechanistic and processing-oriented reviews consolidate how the degree of methylation and the ionic environment set gel strength, brittleness, and syneresis risk [59], offering practical levers for extrusion stability [60]. Reactive and three-dimensional printing studies further highlight acidity (pH) and calcium-ion activity as control variables during deposition, enabling early locking without overly thick shells and expanding the printability window for thin walls and overhangs [61]. In application-driven designs, starch cores encapsulated by alginate–pectin shells achieve acidity-responsive (pH-responsive) release while maintaining strand fidelity, linking formulation to function beyond geometry alone.

The bottlenecks mirror those of alginate but are compounded by the sensitivity of pectin to ionic strength and drift in acidity (pH). Elevated calcium-ion concentrations near the nozzle can trigger premature gelation, higher extrusion pressures, and filament fracture; during storage, unbuffered systems may exhibit syneresis and dimensional change. Mechanistic work on low-methoxyl pectin with internal calcium-ion sources shows that tuning the precursor level and the acidification rate moderates these trade-offs by smoothing gelation fronts and improving recovery after shear [62]. Future work should (i) formalize rheology-to-geometry maps at the build temperature alongside simple geometric readouts to define transferable printability windows; (ii) employ controlled calcium-ion delivery—micro-misted or sprayed, or coaxial low-dose exposure—to avoid over-gelation near the nozzle while retaining rapid setting; and (iii) advance function-aware architectures in which alginate–pectin shells provide graded stiffness and acidity-triggered permeability around starch or protein cores. Together, these steps would translate the biochemical advantages of low-methoxyl pectin into reproducible, shelf-life-aware formulations for edible three-dimensional printing [60].

### 3.4. Kappa-Carrageenan

Kappa-carrageenan (κC) is an effective early yield-stress booster in protein and starch matrices, enabling immediate strand stand-up and limiting creep during the first seconds after deposition [63,64]; both in situ gelation and emulsion-gel formulations demonstrate clean filaments and reliable wall-angle retention when the kappa-carrageenan level and the process temperature are controlled [65,66]. Whey-protein–kappa-carrageenan emulsion gels achieve smooth lines and robust self-support as kappa-carrageenan raises viscosity and gel strength [67], with concentration sweeps indicating an optimal range near approximately 0.6 percent for tall builds [68]. At higher kappa-carrageenan levels or under aggressive cooling, embrittlement, shrinkage, and interlayer delamination emerge, compromising chewability and crack resistance [69]. To buffer brittleness while maintaining early support, kappa-carrageenan is often combined with more elastic modifiers or dispersed phases: konjac glucomannan reduces syneresis and improves elasticity in kappa-carrageenan-based food inks, and oil-in-water emulsion droplets can enhance deposition stability without sacrificing printability [70]. Overall, carrageenan-forward formulations benefit from composition–process co-optimization—kappa-carrageenan dose, thermal profile, and inclusion type—to balance early support with ductility and layer cohesion for reliable, edible three-dimensional structures [71]. Some representative studies on carrageenan gels for 3D food printing are discussed in Table 3.

### 3.5. Agar

Agar exhibits thermally hysteretic gelation, solidifying upon cooling at a relatively high setting temperature and forming a strong gel network that underpins high edge fidelity, sharp corners, and minimal slump at ambient conditions, features desirable for decorative geometries and thin-wall stability in extrusion printing [74]. Comparative evaluations of agar and agarose inks indicate that low to moderate concentrations (approximately three to six percent by mass) already yield stiff, shape-retaining strands while preserving processability when shear is imposed within the nozzle [75]. In food manufacturing workflows, agar is routinely deployed as a room-temperature “lock-in” hydrogel for post-deposition stabilization, complementing hot-melt or warm-extrusion materials and reducing the need for support baths [76].

The principal limitation is brittleness: neat agar fractures readily in bending and exhibits poor interlayer compliance, which can induce microcracks and interlayer debonding in tall builds. Mechanical and rheological studies on agar-rich edible gels quantify high moduli but limited ductility and highlight sensitivity to formulation and to thermal history [77]. To widen the processing window, blending with hydroxypropyl methylcellulose or proteins is effective: hydroxypropyl methylcellulose confers heat-set, thermoreversible behavior and enhances structural recovery after shear, reducing die swell without increasing extrusion pressure when modest syringe-barrel-to-build-plate temperature differentials are applied. Recent semi-solid extrusion studies of agar–hydroxypropyl methylcellulose systems show that small additions of hydroxypropyl methylcellulose soften the network while retaining strand integrity and a surface finish suitable for detailed features [78].

Prospectively, three research directions warrant emphasis. First, establish standardized rheology–geometry mappings at the build temperature—reporting the storage modulus, the loss tangent, and the three-interval thixotropy test together with line-width error, bridge deflection, and wall-angle retention—to substantiate and compare printability claims for agar and agar–hydroxypropyl methylcellulose systems. Second, codify thermal management specified nozzle-to-substrate temperature gradients and rapid post-setting—as an explicit design variable to suppress die swell and modulate interlayer fusion, leveraging agar’s rapid thermally induced gelation without incurring excessive stiffening. Third, prioritize parsimonious, synergistic additives. For example, low-dose hydroxypropyl methylcellulose or food proteins that form plasticizing co-networks—over multi-gum combinations to improve ductility and interlayer cohesion while maintaining formulation transparency.

### 3.6. Starch-Based Gels

Starch systems are moving beyond trial-and-error practice: pregelatinized starch or high-amylose fractions can enhance early strand stand-up and extrudability [79,80,81], while composite starch–protein–hydrocolloid maps provide rational, design-for-process guidance [82,83,84,85]. Storage remains a bottleneck because of retrogradation, syneresis, and dimensional drift; additions of pectin or selected gums stabilize prints stored at low temperatures and maintain texture, pointing to shelf-life-aware formulation [86]. Accordingly, Table 4 summarizes representative starch systems, linking the principal design levers to rheological windows, print fidelity, and storage stability.

Collectively, the studies in Table 4 indicate a shift from empirical tuning to design-for-process. Four levers recur: (i) structure tuning via pregelatinized or high-amylose fractions to increase early stand-up; (ii) network reinforcement through composite starch–protein–hydrocolloid systems—especially kappa-carrageenan (κ-carrageenan) and synergistic xanthan gum with locust bean gum—to increase the storage modulus, decrease the loss tangent, and accelerate structural recovery; (iii) nutrient-dense cereal–legume blends stabilized by small-dose gums to maintain extrudability; and (iv) shelf-life modifiers (pectin and soluble solids) that mitigate retrogradation, syneresis, and dimensional drift during storage at reduced temperatures. These levers consistently translate to higher line fidelity and self-supporting lattices and thin walls, yet notable trade-offs emerge: excessive gum loadings hinder flow and promote nozzle clogging; waxy (amylopectin-rich) bases sag; and overly high amylose interrupts continuous extrusion—implying formulation-specific optima rather than universal recipes. Methodologically, the field benefits from reporting print outcomes alongside rheology at the build temperature and storage behavior. Going forward, standardized geometric metrics (for example, line width error and bridge deflection) and simple process proxies (for example, the die swell ratio) should be coupled with temperature–time histories to link rheological windows to both immediate geometric fidelity and refrigerated stability.

### 3.7. Gellan, Methylcellulose, Xanthan, Konjac, and Blends

Across extrusion workflows, these hydrocolloids function as complementary control knobs acting through orthogonal gelation triggers and network effects. Gellan gum provides a temperature-sensitive setting that arrests die swell and locks geometry early; curing strategies explicitly exploit this phase change to stabilize unsupported features in edible inks [94]. Methylcellulose offers a heat-set mechanism: viscosity is low within the nozzle but rises rapidly upon heating, enabling support-like stabilization and high-fidelity contours in material-extrusion printing [95]. Xanthan gum acts as a network intensifier and recovery accelerator; at low doses—often in synergy with locust bean gum—it decreases the loss tangent, increases apparent strength, and reduces lattice sag, although overdosing penalizes extrudability [90]. Konjac glucomannan enhances self-support in hot-extrusion or composite matrices and improves strand integrity; recent studies report improved print quality when konjac glucomannan is tuned alone or combined with other gums [96].

Representative implementations illustrate these roles. Fish-gelatin and high-acyl gellan-gum inks yield clean filaments and tall features at modest total solids, validating gellan gum as a low-dose structure modulator in protein-rich systems [43]. Methylcellulose hydrogels have been three-dimensionally printed as biodegradable supports or functional matrices, leveraging thermal gelation to maintain shape and minimize deformation during and after deposition [95]. In carbohydrate-rich inks, xanthan gum–locust bean gum synergy strengthens potato-starch gels and jelly formulations, yielding straighter lines, sharper corners, and reduced sag at processing-relevant shear rates [90,97]. Meanwhile, konjac glucomannan improves stand-up under hot-extrusion conditions and enhances the printability of cereal-based gels, supporting layer-by-layer construction of nutrient-dense designs [98].

Taken together, gellan gum (cool-set) and methylcellulose (heat-set) provide early geometry locking; xanthan gum (often with locust bean gum) tunes shear thinning and structural recovery for bead-width control; and konjac glucomannan augments network integrity—especially in protein- or starch-rich blends. Optimal performance typically arises from minimal, synergistic pairs rather than multi-gum stacking, and from pairing geometric metrics (line width error and bridge deflection) with rheology at the build temperature to establish transferable printability windows across platforms [99].

### 3.8. Protein Gels

Protein gels are increasingly used as structure–function scaffolds in extrusion-based edible printing because their gelation pathways—thermal denaturation (heat-set), salt- and acidity-driven aggregation (cold-set), and enzyme-catalyzed crosslinking—allow decoupling of within-nozzle flow from post-setting firmness [100,101,102,103,104]. Heat-induced whey-protein-isolate gels, formulated with controlled ionic strength and fat content [105,106,107], have demonstrated reliable line fidelity and self-support when rheology is tuned at the build temperature, providing a baseline for protein-rich inks [108]. Enzymatic induction adds orthogonal control: transglutaminase-mediated crosslinking increases cohesion and structural recovery, improving layer retention at comparable total solids and enabling milder thermal histories [109]. On the plant side, soy-protein-isolate emulsion gels—often reinforced with galactomannans such as guar gum or xanthan gum—translate effectively to extrusion by co-optimizing interfacial viscoelasticity and bulk network strength, yielding stable lattices with tunable mouthfeel [22,110]. Hybrid matrices broaden the design space: yogurt-based gels combining whey-protein isolate and gelatin achieve high-protein prints that balance edge definition with sensory acceptance, whereas phase-structured whey-protein–gellan-gum systems illustrate how controlled incompatibility can raise stand-up without excessive total solids [111].

Limitations arise from protein chemistry and process sensitivity. Over-aggregation or over-crosslinking increases brittleness and extrusion pressure; salt and acidity windows are narrow, and temperature histories are path-dependent, amplifying the coupon-to-part gap in tall builds. Water migration and syneresis erode dimensional stability during storage at reduced temperatures, particularly in lean networks or emulsion-rich matrices with droplet coalescence. Sensory constraints further bound the feasible rheology. Transferability suffers from heterogeneous reporting of build-temperature rheology and the scarcity of dimensionless descriptors that generalize across nozzles, speeds, and climates.

Future directions should prioritize: (i) co-network strategies with alginate, low-methoxyl pectin, kappa-carrageenan, or gellan gum to raise early yield stress while mitigating brittleness at comparable stand-up; (ii) induction-program design (enzyme dose, ionic strength, and controlled acidity ramps) to coordinate gelation fronts with deposition time; (iii) emulsion-gel engineering (droplet size and oil fraction) to tune ductility without sacrificing line fidelity; and (iv) standardized reporting, the storage modulus, the loss tangent, and structural recovery at the build temperature paired with geometric metrics (line width error, bridge deflection, and wall-angle retention) and simple shelf-life assays (syneresis and shape drift). Embedding these elements into minimal, shelf-life-aware recipes will move protein gels from empirical tuning toward portable, application-aligned formulations for high-protein, texture-modified, and functional foods [112].

### 3.9. Emulsion Gels

Emulsion-structured inks: emulsion gels (protein/polysaccharide matrices trapping oil droplets), high-internal-phase emulsion (HIPE) gels, Pickering emulsion gels, and bigels (coupled hydrogel–oleogel networks) increasingly underpin extrusion prints that require low die swell, ductility, and moisture retention at comparable water activity [113,114,115]. Across recent studies, interfacial design (protein–polysaccharide complexes or particles), oil fraction, droplet size, and the setting pathway (thermal, ionic, enzymatic) jointly tune the yield floor and viscoelastic recovery that govern line width and wall stability. Plant-protein/inulin HIPE gels, for example, deliver stand-up lattices with improved sensory quality and reduced greasiness, while maintaining extrudability through shear thinning and rapid post-shear recovery [116,117]. Bigels expand this idea by co-percolating oil and water networks; mapping oleogel: hydrogel ratio and emulsifier identity yields processable windows where strand continuity and layer cohesion are preserved during tall builds [118,119]. Pickering emulsion gels stabilized by biopolymers or particles offer coalescence resistance under printing shear and retain shape in open lattices [120,121]; probiotic-loaded designs further demonstrate structure–function coupling beyond geometry [122,123]. Authoritative syntheses now codify these levers into design-for-process guidance linking interfacial architecture and network connectivity to printability metrics and release behavior [124].

**Figure 2:**
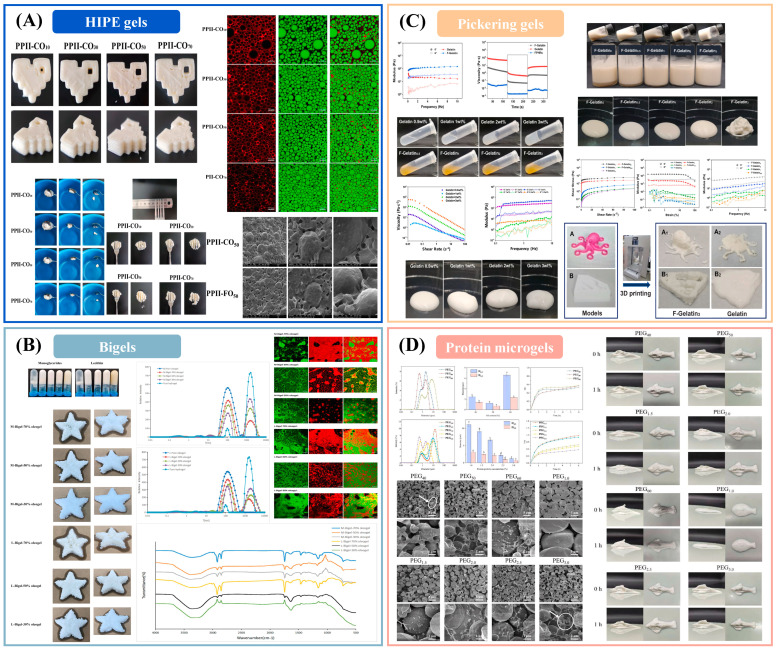
Emulsion-gel inks for extrusion 3D printing. (**A**) Pea protein–inulin HIPE inks [116]; (**B**) Candelilla-wax/gelatin bigels [118]; (**C**) Microalgal residue–gelatin Pickering gels [122]; (**D**) Soy protein microgel Pickering gels [125].

High-internal-phase emulsion inks (approximately eighty-five percent oil by mass) stabilized by pea-protein–inulin complexes achieve reinforced interfacial and bulk viscoelasticity with high thixotropic recovery (Figure 2A), enabling accurate printing and meeting the International Dysphagia Diet Standardization Initiative texture requirements when the inulin level and the oil type are optimized [116]. Composition-tunable bigels comprising candelilla-wax oleogels and gelatin hydrogels form printable architectures in which emulsifier identity and the oleogel-to-hydrogel ratio govern microstructural transitions (Figure 2B), crystallinity, and the controlled co-release of lipophilic and hydrophilic active ingredients [118]. Pickering emulsion gels prepared from Haematococcus pluvialis residue–gelatin complexes provide shear thinning, thixotropic networks at low total solids that yield high-resolution prints and function as structural fat replacers (Figure 2C) [122]. Oil-in-water Pickering gels stabilized by soybean-protein-isolate microgels develop particle-spanning networks with increased viscosity and storage modulus at higher oil and particle contents (Figure 2D), supporting smooth extrusion and high geometric fidelity with scope for active-ingredient delivery and texture tailoring [125].

Despite these advances, emulsion-gel microstructure exhibits strong path dependence on shear and thermal history, complicating extrapolation from coupon-scale tests to full builds. Over-structured matrices—characterized by high interfacial coverage or dense network connectivity—improve early stand-up yet increase extrusion pressure and the likelihood of brittle fracture, whereas under-structured systems display creep, interlayer slip, and delamination. During refrigerated storage, oil leakage or creaming and aqueous-phase migration (syneresis) drive dimensional drift, especially in lean continuous phases or at high dispersed-phase volume fractions. From a formulation standpoint, certain emulsifiers and wax-based structurants may conflict with clean-label constraints or introduce flavor masking. Methodologically, cross-study comparability is limited by inconsistent reporting of rheology at the build temperature and by the infrequent linkage of those measurements to standardized geometric benchmarks (for example, line width error and bridge-deflection coupons) with quantified uncertainty.

Looking ahead, progress in emulsion-structured inks will depend on translating microstructure into transportable process rules. A priority is the construction of microstructure-aware, dimensionless maps—anchored by the Capillary number to balance interfacial stresses against flow, the Yield number or the Bingham number to govern stand-up, and a simple die-swell surrogate—to normalize printability across printers, tool paths, and climates. In parallel, double-network strategies (for example, Pickering interfaces combined with biopolymer gels or bigel architectures) should be leveraged to decouple early support from ductility without resorting to excessive solids loading. Real-time characterization—via in situ optical imaging or optical coherence tomography—should link droplet-scale stability with bead-width control and interlayer welding, thereby closing the loop among formulation, rheology at the build plane, and multilayer fidelity. Finally, routine pairing of geometric metrics with shelf-life assays—coalescence and creaming, syneresis, and dimensional drift during refrigerated storage—will allow printability windows to predict not only immediate strand fidelity but also storage performance and release profiles. Recent syntheses already outline these trajectories and curate formulation exemplars that are well suited for inverse design; adopting such frameworks will shift emulsion gels from empirical tuning to predictable, application-aligned manufacturing.

### 3.10. Clean Label and Consumer Acceptance

Clean label considerations are central to edible hydrogel printing because ingredient lists are highly visible and closely examined by consumers [126]. Formulations should prioritize hydrocolloids and crosslinking triggers that are familiar in foods including pectin, alginate, starches, gelatin, agar, kappa carrageenan, calcium salts, pH adjustment, thermal setting, and transglutaminase [38]. Acceptance improves with short and transparent labels and careful choice of mineral salts that avoid astringency, which aligns with recent evidence on how consumers judge ingredient naturalness and acceptability [127]. When minor additives are required to widen the printability window, such as glycerol or soluble fibers, their levels should be kept low and declared clearly while maintaining rheological targets for fidelity [128]. Beyond composition, consumer expectations of texture and flavor are shaped by strand-based microstructures, so print parameters that influence mouthfeel, including strand diameter, infill, and anisotropy, should be selected to match familiar category cues in personalized products.

### 3.11. Shelf Life and Stability in Printed Gels

Printed geometries present high surface area to volume ratios and many internal interfaces, so their stability constraints differ from cast gels and require explicit reporting in studies of 3D printed foods [129]. Important modes of change include moisture exchange, syneresis, retrogradation or phase separation, microbial growth, oxidative or enzymatic degradation, color drift, and sensory fatigue, and several recent works emphasize quantifying these changes during storage [130]. A concise minimum dataset should include initial water activity and pH baseline microbiology at day zero, mass change and syneresis at defined time points at chilled and intended ambient conditions, retention of texture or viscoelastic properties over storage, color difference, and sensory acceptability at the intended consumption time [131]. Packaging and storage conditions, including temperature, relative humidity, and any use of modified atmosphere, should be specified with reference to current approaches that improve microbial stability [132]. Post-set enablers can be used to improve stability, including ionic or enzymatic crosslinking, mild dehydration, or control of water activity and geometry choices such as thicker strands or lower porosity to slow moisture exchange while maintaining printability [133]. These stability requirements can be integrated with the decision-oriented printability workflow so that formulations are tuned not only for shape fidelity at print time but also for the targeted distribution and use environment.

In hybrid and emulsion-based formulations, the most consequential unknowns for long-term dimensional stability and active-ingredient delivery include droplet coarsening or creaming under temperature cycling, water activity gradients that induce shrinkage or swelling, interfacial layer rearrangements that reduce modulus and cohesion, ion exchange and pH drift that shift network permeability, and the coupling of droplet size distribution with pore geometry that governs diffusion and partitioning.

## 4. Printing Technologies and Post-Set Enablers

### 4.1. Extrusion-Based Deposition

Extrusion has become the default modality for edible gels because it tolerates high-viscosity, particulate [134], and multiphase inks while still enabling flexible tool paths—practically achieving filament continuity and early strand stand-up where drop-on-demand methods fail at comparable total solids [45]. Coaxial printheads further provide early shape locking by delivering a calcium-ion (Ca^2+^) sheath around alginate, low-methoxyl pectin, or starch cores, increasing wall-angle retention and enabling taller builds without increasing total solids or syringe-barrel pressure [25]. Recent metrology links feasible extrusion-speed ranges and pressure–flow calibration directly to rheological model fits, allowing pressure-limited printers to operate closer to their practical envelopes with fewer trial prints [26]. A persistent limitation is elasticity at the nozzle exit and the resulting die swell, which inflates bead width beyond the nozzle inner diameter; experimental and computational fluid dynamics studies show swell scaling with elastic recoil and exit pressure, motivating shorter free-flight distances and earlier locking when thin features are targeted [35]. Screw extrusion widens the operating window by homogenizing shear histories and gently remixing phase-separating inks, but broad residence-time distributions can overheat formulations or induce premature gelation unless thermal profiles are instrumented and controlled [45]. Looking forward, in situ sensing—optical coherence tomography for layer-by-layer geometry and defect maps, and ultrasound for interlayer bonding and gelation state—should close the loop on pressure, speed, and cooling control to suppress die swell in real time [33].

**Figure 3:**
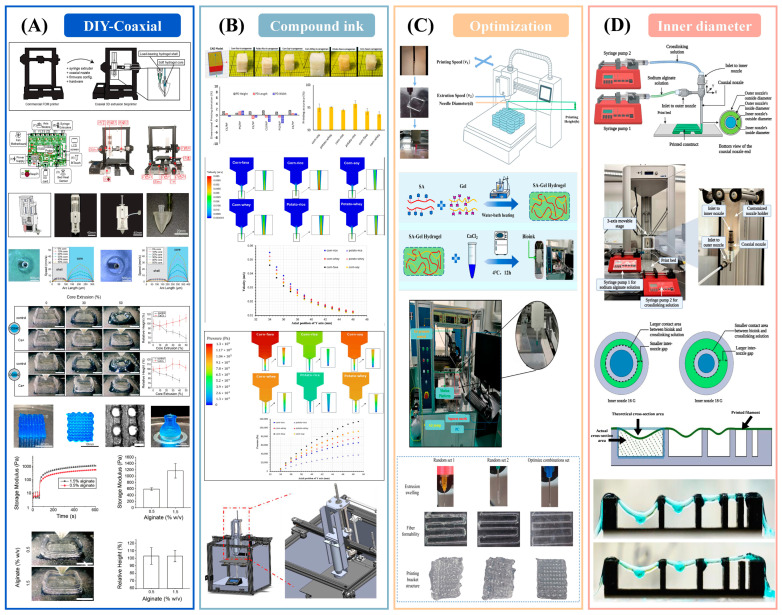
Empirical demonstration of platform-recipe-process-geometry coupling. (**A**) DIY coaxial, viable soft core [135]; (**B**) κ-carrageenan inks, high fidelity [83]; (**C**) Data-driven tuning reduces error [136]; (**D**) Inner nozzle controls crosslinking/strength [137].

Recent experimental studies collectively outline a closed-loop pathway from hardware to formulation and process optimization for extrusion-based deposition. Low-cost, do-it-yourself coaxial platforms have demonstrated stable deposition of “soft-core/load-bearing-shell” architectures using dual syringe pumps and coaxial nozzles; shape fidelity was governed by the core-to-shell flow ratio, nozzle configuration, and in situ crosslinking (Figure 3A), while mesenchymal stem cells maintained high viability in soft alginate–gelatin cores [135]. In food applications, composite starch–protein–hydrocolloid inks revealed a clear correspondence between rheology and geometry (Figure 3B); formulations containing kappa-carrageenan occupied a favorable viscoelastic window—elevated storage modulus and a loss tangent of approximately 0.10 to 0.17—and achieved greater than ninety percent structural fidelity, with Fourier-transform infrared spectroscopy indicating noncovalent strengthening and computational fluid dynamics linking pressure and velocity fields to deposition behavior [83]. At the process level, orthogonal experimental design with gray relational analysis and support-vector-regression combined with particle-swarm optimization targeted filament formability and the die swell ratio (Figure 3C), reducing geometric error relative to naive parameter sets [136]. Under coaxial conditions, the inner nozzle diameter critically affected the efficiency of in situ ionic crosslinking and the resulting filament strength (Figure 3D), revealing a coupling between nozzle geometry, mass transfer, and gelation kinetics [137]. Across printers, calibration records nozzle, barrel, and build surface temperatures and their stability, verifies viscosity at the build temperature, establishes the pressure-to-flow relation for the specific extrusion path, and confirms convergence by matching predicted and measured first layer line width.

To improve transferability and accelerate engineering adoption, methods should advance from measurement to decision-making. A minimal, standardized benchmark and metric set—including line-width error—is proposed, the area fraction of over- and under-extrusion, bridge deflection, fusion width, layer thickness, and the die swell ratio—paired with rheology reported at the build temperature (apparent yield stress, storage modulus, loss tangent, and the three-interval thixotropy test). Dimensionless groups should be used routinely to normalize across nozzles, speeds, and environments. Modeling should progress from steady computational fluid dynamics to unsteady, coupled reaction–transport–flow frameworks that resolve calcium-ion (Ca^2+^) gradients, interfacial diffusion, and thermal and moisture histories. In situ sensing—computer vision, optical coherence tomography, and ultrasound—ought to be integrated into feedback control, supported by calibration ladders, open code, and raw data to enable round-robin reproducibility. Finally, quality metrics must be aligned with the application: bioprinting should extend beyond short-term cell viability to long-term phenotype and mechanical maturation, whereas food printing should map geometric fidelity to sensory attributes, nutrient release, and shelf life.

### 4.2. Drop-on-Demand Inkjet of Precursors

Inkjet printing provides high-resolution dosing and multi-ingredient patterning for low-viscosity precursors, enabling sub-bead features and precise spatial flavoring or micronutrient placement that extrusion often cannot achieve [138]. Its core constraints are viscosity and nozzle health: most edible gels exceed permissible viscosities at the build temperature, so practical routes rely on precursors with post-setting triggers (thermal, ionic, or enzymatic) while avoiding satellite droplets or coffee-ring artifacts [139]. Emerging strategies—mild printhead heating, enzyme-assisted inks, and microgel carriers—expand the usable viscosity range while maintaining stable drop formation, but systematic maps that link precursor rheology, trigger kinetics, and final geometry remain scarce for food-grade systems [31]. In the near term, pairing inkjet printing for surface detail with extrusion for load-bearing geometry, coordinated through synchronized post-setting protocols, is the most robust path to consistent adhesion and dimensional accuracy.

### 4.3. Binder Jetting on Food Powders

Binder jetting decouples shape formation from hydrated-gel rheology, enabling ambient fabrication of porous, crispy matrices and intricate shells, and supporting subsequent infusion or glazing—useful for snacks and decorative structures. Recent studies also demonstrate nutrient fortification with precise dosing using binder jetting, pointing to functional snacks in which geometry and composition can be tuned together [140]. The dominant limitations are low “green” strength and moisture sensitivity: printed bodies often require infiltration, glazing, or co-baking to withstand handling, and water-activity gradients can warp parts unless moderated with barriers or humectants. Future progress hinges on formal powder-property and droplet–powder-interaction maps and on standardized post-treatment schedules so parameters translate across starch–sugar blends and machine platforms [141].

### 4.4. Laser Processing of Sugars/Isomalt

Laser sintering and laser melting of sugar or isomalt deliver crisp, high-definition shells and visually striking lattices that integrate well with soft hydrogel cores—an avenue for desserts and encapsulated fillings when powder properties and thermal exposure are controlled [142]. The design space remains narrow because ingredient thermal tolerance limits inclusions, and caramelization or stickiness can occur; recent equipment and technology reviews confirm the viability of selective laser sintering for food powders but emphasize tighter raw-material specifications than those used in extrusion [143]. The most promising path is process integration rather than new chemistries: matching shell porosity and thickness to hydrogel rheology, coordinating temperature and water-activity gradients during assembly, and laser-texturing inner surfaces to promote shell–gel adhesion without over-melting.

### 4.5. Ionic/Thermal/Dehydration Pathways and Shelf-Life Control

Post-setting converts a printable flow state into a stable architecture. Ionic routes—coaxial exposure, spray application, or brief baths—provide rapid strand stand-up and bridge stability for alginate or low-methoxyl pectin cores at modest total solids, but excessive ion flux yields brittle skins and steep core–shell gradients; diluted sheaths and confined exposure lengths mitigate these issues [25]. Thermal routes exploit gel-specific hysteresis—cooling for gelatin, agar, and kappa-carrageenan; heating for methylcellulose—to shorten recovery relative to inter-pass dwell time without increasing total solids, but narrow operating windows and uneven fields can cause clogging or uneven adhesion unless syringe-barrel and build-plate profiles are logged and controlled [139]. Dehydration increases strength and reduces ooze in service yet risks shrinkage and staling; pectin additions and gel-specific modifiers improve stability during storage at reduced temperature and dimensional retention in starch-rich systems, arguing for shelf-life-aware formulation and quantitative drift tests [86]. Downstream packaging and water-activity control, as well as hydrogel-based active packaging, can further extend shape and bite, although standardized biodegradation and shelf-life protocols are still needed [144]. Looking forward, standardizing reports of calcium-ion concentration and flow rate, temperature fields, and dehydration schedules—paired with in situ optical coherence tomography and ultrasound to verify shell formation and interlayer bonding—will enable reproducible early locking across platforms and will feed surrogate models for tool-path and post-setting co-optimization [33]. For ionic and gelation protocols, calibration reports ion activity and exposure length, expresses exposure using a sheath-to-core flow ratio together with nozzle diameter and travel speed as a dimensionless group, limits gradients that lead to shell brittleness, and declares convergence when line width error and bridge deflection fall within predefined bounds.

### 4.6. Technology Comparison and Post-Set Enablers

To bridge the narrative discussion with practical options, this subsection provides a concise, evidence-based overview of the main food printing process routes and their ultimate triggers. Table 5 presents some representative studies and lists deposition principles, practical material windows, resolution and throughput metrics, compatible post-processing strategies, advantages and limitations, and typical food applications. The goal is not to recapitulate the complete approach, but rather to translate recent findings into decision-oriented guidance to complement the printability workflow and printability atlas.

## 5. Gel-Forward Product Applications

### 5.1. Therapeutic Soft Gels and Low-Sugar Desserts

Extruded gels based on gelatin with kappa-carrageenan, low-methoxyl pectin with alginate, and emulsion-gel matrices already meet the International Dysphagia Diet Standardization Initiative while retaining recognizable forms. Coaxial calcium-ion (Ca^2+^) delivery stabilizes walls and overhangs in low-sugar fruit and dessert builds, providing rapid structural recovery at modest total solids; light post-dehydration tightens the surface “skin” for sharper edges. Fragility arises when ionic skins are over-hardened, producing brittle bites and cracking, whereas unbuffered systems drift in acidity (pH) and ionic balance and weep during storage at reduced temperatures, distorting geometry. A pragmatic consolidation is to standardize an IDDSI-aware printability panel: report apparent yield stress, storage modulus, and loss tangent at the build temperature, together with three calibration coupons (single line, fixed-span bridge, and thin wall); log water activity, dimensional drift, and exudate mass over forty-eight to seventy-two hours. Buffered, diluted calcium-ion delivery with confined exposure (coaxial or micro-spray) minimizes gradients; low-methoxyl-pectin/alginate shells provide early locking; and emulsion droplets or waxy starches temper shrinkage during storage at reduced temperature while preserving flavor.

### 5.2. Structured Savory Foods and Emulsion-Gel Carriers

Plant-based lattices and graded textures stand up when kappa-carrageenan, xanthan gum, or konjac glucomannan elevate early yield stress, and when gellan gum or methylcellulose provides thermal locking. Emulsion-gel and bigel matrices—including high-internal-phase emulsion and Pickering architectures—reduce die swell, stabilize bead width, and add juiciness with co-delivery of lipophilic and hydrophilic active ingredients; these strategies improve first-layer fidelity and multilayer cohesion at comparable water activity. Limits arise from batch-variable plant proteins, degradation during reheating and freeze–thaw cycling, microstructural path dependence that decouples coupon-scale rheology from build outcomes, and sensory masking from certain emulsifiers or wax-based structurants. Consolidated practice is to decouple early geometry from final chew using double networks (an interfacial scaffold combined with a bulk gel), extend the metric set with extensional and adhesive tests for reheating robustness, and normalize across printers and climates by reporting the Capillary number, the Yield or Bingham number, and the die swell ratio alongside classic print metrics. Favor clean-label particles or protein–polysaccharide complexes at minimal doses, and co-report thermal or ionic setting kinetics with geometry coupons to make results transferable.

### 5.3. Hybrid Shell–Core Architectures

Crisp sugar or isomalt shells fabricated by laser processing or binder jetting pair effectively with soft hydrogel cores to deliver striking crunch–soft contrasts and precise micronutrient dosing while decoupling complex shape from hydrated-gel rheology. Success depends on matching shell porosity and thickness and inner-surface roughness to core rheology, and on timing assembly within narrow thermal and moisture windows. Failure modes cluster around mismatched water activity, warping, weeping, weak green strength, and variable adhesion when post-processing is improvised. A unified playbook publishes powder-property and droplet–powder-interaction maps with fixed post-treatment schedules, instruments water-activity gradients during assembly and early storage, and uses hydrophobic barriers or fat-phase glazes before hydrogel infusion; routine sweating tests and adhesion checks tighten quality control without sacrificing throughput.

### 5.4. AI-Enhanced Formulation–Process–Quality Closed Loop

**Figure 4:**
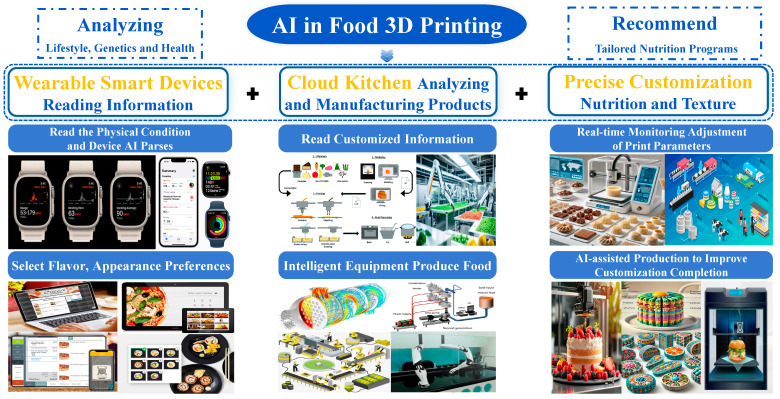
AI-Orchestrated Closed Loop for Personalized 3D Food Printing.

Figure 4 depicts a “wearables → cloud kitchen → precise customization” chain as the backbone, in which artificial intelligence (AI) converts personal health and preference data into printable gel formulations and closes the loop during manufacturing and quality control. On the front end, smartwatches, bands, and home-health devices supply longitudinal signals—heart-rate variability, sleep quality, physical activity, post-prandial glucose, and medical restrictions—overlaid with preferences for flavor, appearance, and mouthfeel. Multimodal models translate these inputs into nutritional and sensory targets (macronutrients and micronutrients; texture and swallowing safety such as IDDSI levels; and desired geometry and surface detail). These targets are then rendered into printability thresholds and process windows at the build temperature, apparent yield stress, storage modulus, loss tangent, and recovery measured by the three-interval thixotropy test, tied to geometric metrics such as line width error, bridge deflection, wall-angle retention, and fusion width.

In the cloud-kitchen layer, artificial intelligence (AI) performs a dual task of inverse design and process orchestration. Leveraging standardized formulation–rheology–geometry triplets from prior experiments, active learning or Bayesian optimization rapidly identifies compositions that meet apparent yield stress, storage modulus, loss tangent, and three-interval thixotropy test targets with minimal screening, particularly for protein–polysaccharide co-networks, emulsion gels, and bigel or double-network systems. Gray-box process models map pressure, speed, nozzle inner diameter, layer height, and—when used—coaxial calcium-ion (Ca^2+^) flow rate to the die swell ratio, interlayer adhesion, and edge definition, generating first-shot parameter sets within tolerance. For builds that require early lock-in, the path planner schedules localized calcium-ion exposure or cooling so that geometric fidelity is decoupled from the final bite.

On the manufacturing floor, “precise customization” is realized through real-time sensing and adaptive control. Cameras, scales, and simple ambient sensors stream layer images, mass, and thermo-hygrometric conditions; lightweight convolutional neural networks (CNNs) and time-series models detect over- and under-extrusion, delamination, voids, and premature skinning with interpretable labels. Controllers then adjust pressure, speed, thermal set points, and ionic dosing to keep line width, die swell, and bridge sag within bounds. For multimaterial or shell–core architectures, the system jointly manages shell porosity and thickness, inner-surface roughness, and core gelation kinetics to secure adhesion and shape while preserving crisp–soft contrasts. Post-print logs over twenty-four to seventy-two hours—water activity, dimensional drift, and exudate mass—feed back to the cloud to train shelf-life and sensory-decay predictors that screen out formulations which print well but store poorly.

To make the depicted loop portable across kitchens and climates, data and models must be standardized and governed. The minimal record should include build-temperature rheology, device and process states, measurements on three benchmark artifacts (single line, fixed-span bridge, and thin wall), and early-storage descriptors. Inputs are best normalized with dimensionless groups—the Capillary number, the Yield number or the Bingham number, and the die swell ratio—to reduce cross-printer variability. Each AI-proposed formulation should ship with a model card stating allergen and labeling implications, safe operating bounds, and intended populations; health-related deployments should retain human-in-the-loop sign-off by dietitians or chefs. Privacy and safety require on-device preprocessing of wearable-device data and privacy-preserving network links so the cloud retains only minimal statistics for learning; in the event of model drift or sensor loss, the system reverts to conservative default formulations and process windows to keep food safety first.

Through this sense-plan-execute-learn loop, AI does more than accelerate trial-and-error: it orchestrates personal nutrition goals, the material constraints of printable gels, and manufacturing-floor variability within a single control framework, increasing first-pass yield and storage stability and moving personalized diets from demonstration to scalable delivery. In the near term, real-time feedback optimized printing is expected to mature in centralized kitchens and industrial lines with integrated sensing and quality assurance, whereas home use will rely on a semi-closed loop mode based on validated recipe and parameter recommendations with camera-based checks and conservative bounds on pressure, speed, and temperature. Operationalization from laboratory studies to pilot lines must account for practical constraints across materials, processes, and compliance. Primary risks to rapid translation from laboratory demonstrations to pilot manufacturing include raw material variability and phase migration that shift rheology and shelf life, insufficient calibration and in-process monitoring that impair control of line width and interlayer bonding, and regulatory requirements for ingredient labeling, allergen control, traceability, and stability evidence for active components.

### 5.5. Nutritional Functionality and Sensory Performance

Printed gels can act as delivery matrices that protect and release bioactives in a controlled manner. Release kinetics can be tuned by adjusting diffusion path length through strand diameter and infill density, by tightening or loosening the network reflected in storage modulus G′, and by selecting post-set triggers that form bonds at mild conditions such as ionic or enzymatic routes. Strategies that raise zero shear viscosity and slow structural breakdown can increase gastric residence time and perceived fullness, while co-printing soluble fibers or designing lamellar infill that hydrates slowly can moderate the rate of starch accessibility and help manage the postprandial glycemic response. These choices are most effective when linked to the decision-oriented printability workflow so that measurable gates such as yield stress, recovery, and strand fusion are used to balance nutritional targets with geometric fidelity and throughput in real food settings.

Flavor release and mouthfeel in printed gels arise from partitioning within the matrix and from the creation of fresh surface during oral processing. Geometry choices that increase surface renewal, such as thin strands or porous infill, can accelerate aroma release, while stronger and more cohesive networks can retain volatiles for longer but may mute top notes. Mouthfeel depends on cohesiveness, adhesiveness, and fracture behavior, and printed anisotropy can be used to guide chew down and melt in the mouth toward the expectations of a given category. Sensory evaluation should be aligned with the geometry that will be consumed, and results should be interpreted alongside instrumental texture or viscoelastic metrics so that sensory targets are reconciled with printability constraints and with the same decision-oriented workflow used for formulation and process selection.

## 6. Conclusions

Edible gels are not passive carriers but the primary design lever in three-dimensional food printing. Treating the gel network as the operative unit links microstructure to performance: build-temperature rheological gates—apparent yield stress, storage modulus, loss tangent, and three-interval thixotropy (structural recovery)—predict filament continuity, strand stand-up, wall-angle retention, interlayer fusion, and die swell. When these gel metrics are paired with standardized calibration artifacts (single line, fixed-span bridge, and thin wall) and image-based error metrics, printability windows become portable across platforms. Progress is strongest where gel chemistry provides early lock-in without high total solids: low-methoxyl pectin with alginate; kappa-carrageenan with xanthan gum or konjac glucomannan; gellan gum or methylcellulose thermal sets; and emulsion-structured gels—high-internal-phase emulsion, Pickering, and bigels—that add ductility, moisture retention, and controlled release at comparable water activity. Because gel microstructure is path dependent, coupon-scale rheology must be interpreted alongside recorded thermal and ionic histories and in-process sensing. Shelf life is a gel property as well: routine logs over twenty-four to seventy-two hours of water activity, dimensional drift, and exudate mass ensure that printed objects retain gel integrity and stable geometry.

Normalization with gel-relevant dimensionless groups resolves cross-printer and climate variability. A concise checklist—gel rheology at the build temperature; calibrated pressure–speed–flow relations; standardized artifact scores; explicit thermal setting and dehydration histories; and early-storage logs—enables artificial-intelligence (AI)-assisted inverse design and gray-box control under human oversight. With gels centered, the field moves from descriptive demonstrations to predictive, regulation-ready manufacturing of personalized, nutritious, and aesthetically precise gel-forward foods.

## Figures and Tables

**Table 1:** Quantitative evaluation of print quality: Indicators, benchmark components, and representative studies.

Research Topic/Material and Platform	Quantitative “Print Quality” Metrics	Benchmark Artifact/Condition	One-Sentence Key Conclusion	Reference
Automated image assessment of intra-/inter-layer geometric accuracy (food extrusion)	Over-/under-extrusion area %, strand-diameter error, layer infill ratio	Straight line–corner–honeycomb/rectangular infill; deer-shaped example	Digital image analysis agrees closely with human scoring and provides correction guidance.	[28]
Image + machine learning to predict printability of edible polysaccharide inks	Strand diameter and surface roughness; printability classification via RSM/ML	Straight line and standard line segment	Diameter and roughness alone effectively separate printable vs. non-printable formulations.	[29]
Vision-feedback path compensation (bio/hydrogel extrusion)	Path–filament deviation Δxy, contour error	Straight lines, arcs, grids	Real-time vision compensation significantly reduces contour error and improves repeatability.	[30]
Texture analyzer as a surrogate for rheometer in printability classification (gummy system)	Equivalent viscosity/yield parameters → print pass/fail	Single line/thin wall	Texture analysis approximates key rheological information, enabling rapid formulation screening.	[31]
Agar–HPMC printability and rheological window	G′/tan δ, apparent viscosity → line-width error, collapse	Single line, thin-walled column	Cold-gelation trigger plus compatibilization achieves high line fidelity at low solids.	[32]
Extrusion speed/flow-rate metrology for parameter optimization	Measured volumetric flow rate, line-width error, speed–pressure calibration	Varied nozzles and speed settings	Speed–flow calibration markedly tightens the line-width prediction interval.	[26]
In-situ ultrasound monitoring of hydrogel printing	Acoustic readouts of layer thickness/interfacial bonding and elastic evolution	Continuous extrusion + Ca^2+^ curing	Subwavelength ultrasound detects layer defects online and guides curing timing.	[33]
In-situ OCT measurement of multi-material printing accuracy	Strand diameter, layer thickness, printhead synchronization error	Co-fabrication of multiple materials	OCT-based closed-loop control reduces deposition error at material interfaces.	[34]
Experimental/numerical measurement of extrusion die swell	Exit strand-to-nozzle diameter ratio. Velocity dependence	Varied temperature/speed	Sr serves as a geometric proxy for exit elastic recoil and correlates with line-width drift.	[35]
Design-feature-based printability scoring	Corner radius, bridge deflection, overhang success rate, line-width error	Single line, 90° corner, fixed-span bridge, open-cell lattice	DfAM-style scoring enables rapid comparison across formulations and toolpaths.	[27]
Machine-learning-driven hydrogel printability database	Horizontal/vertical geometric error ↔ HB/3ITT features	Standard line/wall	A 150-case dataset yields transferable predictive relationships across formulations.	[36]
Computer-vision metrology of instantaneous extrusion rate/strand width	Instantaneous extrusion rate; time-varying line width	Pressure-stabilized extrusion	CV metrology provides a baseline for pressure–speed closed-loop control and under/over-extrusion diagnosis.	[37]

**Table 2:** Application of gelatin ink in extrusion 3D printing.

Focus/System	Key Result	Print Outcome	Trade-Off	Reference
Gelatin + κ-carrageenan composite	Composite improves thermal stability and printability	Complex shapes printed with fidelity	High κ-C may cause syneresis/brittleness	[42]
Fish gelatin + high-acyl gellan (edible ink)	Co-network boosts G′/τy at modest solids	Cleaner filaments; taller features	Overdosing on gellan risks brittleness	[43]
Gelatin-methacryloyl + gellan	Yield stress identified as dominant predictor	Better stand-up and wall fidelity	High τ_y_ increases extrusion burden	[46]
κ/ι-carrageenan gels (cation effects)	Cation type modulates gelation and mechanics	Guides co-network ion tuning	Ionic sensitivity; drift risk	[47]
Gelatin low-temperature deposition	Cooling stabilizes flow and early geometry	Reduced die-swell; cleaner lines	Over-cooling can clog nozzles	[48]

**Table 3:** Representative studies of carrageenan-based gels for 3D food printing.

System/Focus	Key Finding	Print Outcome	Trade-Off/Note	Reference
κ-carrageenan (κC) emulsion gels with sunflower oil	κC emulsion gels remain printable up to 40% oil; layering is evident between printed filaments	Smooth lines; stable cuboids under common settings	Some delamination between layers after compression	[66]
Whey-protein–κC emulsion gels	κC increases viscosity and mechanical strength, improving deposition	Straighter strands; enhanced self-support	Excess κC tends to embrittle the matrix	[68]
Whey-protein–κC emulsion gels	Optimal κC ≈ 0.6% yields best printing performance	Smooth lines; tall builds with good retention	Higher κC reduces ductility and chew	[72]
κC solutions with in-situ gelation	Temperature-triggered gelation supports “print-then-set” strategy	Immediate stand-up; reduced sag	Requires tight thermal control	[65]
κC–konjac glucomannan blends	KGM reduces syneresis/brittleness and improves elasticity of κC gels	Better layer cohesion; fewer cracks	Composition must be re-tuned for shape fidelity	[70]
κC food gels enriched with lupin callus	Inclusion does not preclude printability; texture/digestibility tunable	Printable at several inclusion levels	Matrix heterogeneity may affect layers	[73]

**Table 4:** Starch-Based Gels for Extrusion 3D Printing: Design Levers, Process Windows, and Print Outcomes.

Starch System/Ink	Design Lever	Rheology/Process Notes	Print Outcome	Reference
Normal corn starch + pregelatinized high-amylose + soy/whey proteins	Pregelatinized/high-amylose boosts early support; proteins aid line retention	Wider self-support window; no support bath needed	Fine lines and hollow/overhang structures hold	[82]
Composite starch–protein–hydrocolloid (κ-carrageenan/xanthan/CMC/arabic gum)	Mapping defines printability window; κ-carrageenan G’ rise and tan δ decline	Reported G′ > 4000 Pa, tan δ = 0.096 – 0.169; accuracy 93–96%	High-fidelity lines/lattices; low contour error	[83]
Potato starch + pectin (cold storage)	Pectin mitigates retrogradation/syneresis, limits drift	Better G′/texture after refrigeration; less geometric change	Improved shape retention post-chill	[86]
Corn starch (varying amylose/amylopectin)	Amylose raises support; too high breaks extrusion	Best ratio gave 88.12% accuracy; waxy continuous but weak	Stable lines/thin walls at optimal ratio	[87]
Potato starch + pectin (heating T × pectin)	Heating modulates pectin effect	80–90 °C: G′ rise /viscosity/printability; 70 °C: opposite	Straighter lines/walls/lattices at higher T	[88]
Cereal–legume (germinated brown rice + red adzuki) + xanthan/guar	Gums improve viscoelasticity/printability in nutrient-dense blends	Higher stability; tunable texture	Stand-up walls/thin lattices; fewer collapses	[89]
Potato starch + xanthan/locust bean gum	XG–LBG synergy strengthens network, faster recovery	Viscoelasticity rise; shear thinning with quick recovery	Continuous lines; less lattice sag	[90]
Starch gels with/without κ-carrageenan (multi-source)	High-amylose + κ-carrageenan widens process window	—	High-fidelity lattices/thin walls across cases	[91]
Xanthan gum in starch systems (corn/rice)	XG boosts strength/shape; excess hinders extrusion	tan δ and strength tunable by XG level	Straighter lines; sharper corners	[92]
Rice-flour starch + sucrose	Soluble solids tune viscosity/recovery	Viscosity/recovery modulated by sugar	Smoother surfaces; steadier line width	[93]

**Table 5:** Comparative overview based on one post-2018 study per technology.

Technology	Principle	Materials and Operating Window	Post Set Enabler	Advantage	Limitation	Reference
Pneumatic direct ink writing	Pressure-driven extrusion	Yield-stress alginate or pectin gels; lines about 1 mm; 0.1 to 5 mL per minute	Calcium ionic set by bath or spray	Simple and versatile	Strand swelling and nozzle clogging with particulates	[38]
Screw driven extrusion	Positive displacement screw	High-viscosity particulate pastes; lines about 0.6 to 1.2 mm	Thermal, ionic, or enzymatic at mild conditions	Handles solids with stable metering	Heavier tool head and cleaning load	[145]
Drop-on-demand inkjet	Discrete droplet ejection	Low-viscosity viscoelastic liquids; dots about 50 to 200 micrometers	Ionic spray or enzyme trigger	Very fine patterning	Narrow printable window	[146]
Hot melt extrusion	Melt deposit then cool	Chocolate and sugar melt; filaments about 0.2 to 0.8 mm	Cooling or tempering	Smooth finish with good adhesion	Tight temperature control needed	[147]
Binder jetting	Liquid binder on powder bed	Sugar or starch powders; voxels about 200 to 500 micrometers	Drying or glazing for strength	Complex support-free shapes	Porous parts and limited powders	[148]

## Data Availability

No new data were created or analyzed in this study.
